# Multiple origins and the population genetic structure of *Rubus takesimensis* (Rosaceae) on Ulleung Island: Implications for the genetic consequences of anagenetic speciation

**DOI:** 10.1371/journal.pone.0222707

**Published:** 2019-09-19

**Authors:** JiYoung Yang, Jae-Hong Pak, Masayuki Maki, Seung-Chul Kim

**Affiliations:** 1 Department of Biology, Research Institute for Dok-do and Ulleung-do Island, Kyungpook National University, Daegu, Gyeongsangbuk-do, Korea; 2 Botanical Gardens, Tohoku University, Sendai, Miyagi, Japan; 3 Department of Biological Sciences, Sungkyunkwan University, Suwon, Gyeonggi-do, Korea; National Cheng Kung University, TAIWAN

## Abstract

To determine the origin and genetic consequences of anagenesis in *Rubus takesimensis* on Ulleung Island, Korea, we compared the genetic diversity and population structure of *R*. *takesimensis* with those of its continental progenitor *R*. *crataegifolius*. We broadly sampled a total of 315 accessions in 35 populations and sequenced five noncoding regions of chloroplast DNA. *Rubus takesimensis* emerged as nonmonophyletic and several geographically diverse continental populations were likely responsible for the origin of *R*. *takesimensis*; the majority of *R*. *takesimensis* accessions were sisters to the clade containing accessions of *R*. *crataegifolius*, primarily from the Korean peninsula, while rare accessions from three populations shared common ancestors with the ones from the southern part of the Korean peninsula, Jeju Island, and Japan. A few accessions from the Chusan population originated independently from the Korean peninsula. Of 129 haplotypes, 81 and 48 were found exclusively in *R*. *crataegifolius* and *R*. *takesimensis*, respectively. We found unusually high genetic diversity in two regions on Ulleung Island and no geographic population structure. For *R*. *crataegifolius*, two major haplotype groups were found; one for the northern mainland Korean peninsula, and the other for the southern Korean peninsula and the Japanese archipelago. Compared with populations of *R*. *crataegifolius* sampled from Japan, much higher haplotype diversity was found in populations from the Korean peninsula. The patterns of genetic consequences in *R*. *takesimensis* need to be verified for other endemic species based on chloroplast DNA and independent nuclear markers to synthesize emerging patterns of anagenetic speciation on Ulleung Island.

## Introduction

On oceanic islands, approximately 75% of the insular endemic plant species have originated presumably via cladogenesis, which is typically associated with the term adaptive radiation in insular setting [1,2]. This mode of speciation involves a founding population splitting into several phenotypically and ecologically distinct lineages that can be recognized at a species (or even generic) level; consequently, the initial founding gene pool is partitioned and subjected to intense selection in different habitats, resulting in a low level of genetic variation within each species [1,3]. To reveal and elucidate this mode of speciation, numerous independent research groups have focused on a diverse assemblage of endemic plants on several archipelagoes, such as Hawaii and the Galápagos in the Pacific [4–7] and the Canaries in the Atlantic [8–13]. Also, similar interests have been focused on relatively smaller oceanic archipelagos, such as the Juan Fernández Islands [14–17] and the Bonin Islands [18–19]. The remaining 25% of island endemic plant species, however, likely are derived via anagenetic speciation [1]. This mode of speciation involves an initial founding population on an island, which gradually accumulates genetic variation by mutation and recombination and eventually, without any splitting events, transforms into morphologically and genetically distinct taxonomic forms [1,2]. Unlike the numerous examples of adaptive radiation, the mode of anagenetic speciation on oceanic islands have recently been investigated by a few research groups, primarily in the Juan Fernández Islands in the Pacific [17,20–22] and Ulleung Island in East Asia [23–26].

Ulleung Island, an oceanic volcanic island in the East Sea/Sea of Japan, is located about 137 km off the east coast of the Korean peninsula. The age of the island is approximately 1.8 million years old (myo) and has never been connected to the adjacent continents [27]. A high level of endemism in oceanic archipelagos, such as the Hawaiian Islands (828 species) and the Canary Islands (429 species) is often associated with cladogenetic speciation (95% for Hawaii and 84% for the Canaries), but Ulleung Island is known for its exceptionally high level of anagenetic speciation (33 species; 88% of the total endemic species) [1]. It has been hypothesized that low-elevation islands with low habitat heterogeneity are highly correlated with high levels of anagenetic speciation [1,26]. As of today, the outstanding studies focused on anagenetic speciation on Ulleung Island have been limited to two core eudicot families, i.e., *Dystaenia takesimana* (asteroid II, Apiales, Apiaceae) [23] and *Acer okamotoanum* and *Acer takesimense* (rosid II/Malvidae, Sapindales, Sapindaceae) [24,25]. In a recent review of anagenetic speciation in the endemic angiosperms of Ulleung Island, Takayam et al. [26] summarized that, unlike some previous results based on isozyme studies, AFLP (amplified fragment length polymorphisms) and SSR (simple sequence repeats) molecular markers revealed much higher levels of genetic variation in some species, highlighting the need for the appropriate use of molecular markers. They also pointed out that the anagenetically derived species on Ulleung Island is expected to show high levels of genetic variation similar to (or even higher than) those of the continental progenitor species. Furthermore, given low habitat heterogeneity, no geographic partitioning of the genetic variation owing to gene flow among subpopulations is expected over the landscape [26]. Although these expected genetic consequences of anagenetic speciation on the Ulleung Islands are supported by the results of the few aforementioned studies, we know very little about whether similar patterns can be found in other diverse endemic angiosperm lineages, hindering a synthesis of any emerging patterns on Ulleung Island.

Of nearly 40 endemic species on Ulleung Island, the family Rosaceae (rosid I, Fabidae) includes the greatest number of endemic species (i.e., *Cotoneaster wilsonii*, *Potentilla dickinsii* var. *glabrata*, *Prunus takesimensis*, *Rubus takesimensis*, and *Spiraea insularis*). However, the lack of detailed population genetic studies based on broad samplings of both continental progenitors and anagenetically derived insular species hampers any generalizations regarding the genetic consequences of endemic species-rich angiosperm lineage. In this study, we determined the genetic consequences of anagenetic speciation using a species pair: an insular endemic *Rubus takesimensis* Nakai on Ulleung Island and a continental progenitor *R*. *crataegifolius* Bunge, based on broad geographical sampling and chloroplast DNA sequences. The genus *Rubus*, with worldwide distribution, is a very complex and challenging group owing to agamospermy, hybridization, and polyploidy [28,29], and regional variations in morphology [30–32]. Most species of *Rubus* in Korea belong to the subgenus *Idaeobatus*, and the phylogenetic relationships among them were preliminarily assessed [33,34]. Of approximately 21 species of *Rubus* recognized in Korea, *Rubus takesimensis* is the only endemic species on Ulleung Island, which is morphologically most closely related to *R*. *crataegifolius* found in northeastern Asia (Korea, Japan, China, and the Russian Far East) [34,35]. *Rubus takesimensis*, the insular derivative species, differs from the continental progenitor *R*. *crataegifolius* in its lack of prominent prickles on the stems (i.e., an example of a loss of a defense mechanism) and a general trend towards increasing leaf and flower size (i.e., an example of insular gigantism). Molecular phylogenetic studies established the progenitor-derivative species relationship, allowing us to use them as a model system to study anagenetic speciation on Ulleung Island [34,36,JY Yang unpublished data]. Our preliminary study based on limited insular derivative populations provided some possibilities of multiple introductions of *R*. *takesimensis* on Ulleung Island [36]. This study, however, was based on a very limited sampling of *R*. *takesimensis* (i.e., 37 individuals) and one variable noncoding region of cpDNA (i.e., *trn*L-*trn*F; a total of 391 aligned sites). The initial sampling strategy of the previous study was to broadly sample the continental progenitor species, *R*. *crataegifolius*, to determine the source populations of *R*. *takesimensis*. Therefore, in this study, we made special efforts to broadly sample *R*. *takesimensis* on Ulleung Island (a total of 106 individuals from 12 populations) and also to sample other highly variable noncoding sequences of cpDNA (a total of 4,212 aligned sites). Specifically, we attempted to (1) determine the geographic origin of *R*. *takesimensis*, (2) compare levels of genetic diversity between continental progenitor and insular derivative species and the degree of genetic differentiation between them, and finally (3) examine population genetic structures within insular derivative species. The results are expected to allow us to synthesize any emerging patterns in the genetic consequences of anagenetic speciation on Ulleung Island.

## Materials and methods

### Plant materials

To determine the geographic origin of *R*. *takesimensis* and reveal the genetic structure among populations on Ulleung Island, we sampled the continental progenitor species *R*. *crataegifolius* throughout East Asia (i.e., the Korean peninsula and Jeju Island, China, the Japanese archipelago, and the Russian Far East) and the insular derivative species *R*. *takesimensis* throughout its entire range on Ulleung Island. There were no permit requirements to collect both species in east Asia. For *R*. *crataegifolius*, we sampled a total of 23 populations (209 individuals), including 14 populations (135 individuals) from Korea, 6 populations (51 individuals) from Japan, 1 population (8 individuals) from China, and 2 populations (15 individuals) from the Russian Far East (Table 1). The number of individuals per population ranged from 3 (ND, Chungcheongnam-do, Korea) to 13 (DH, Gangwon-do, Korea). In the case of *R*. *takesimensis*, we sampled a total of 12 populations (106 individuals) throughout Ulleung Island. The number of individuals per population ranged from 7 (THR) to 13 (JR). All individuals were sampled at least 10 m apart after considering the possibility of clonal reproduction in two species. Thus, we sampled a total of 35 populations (315 individuals) for our study species, and *R*. *trifidus* (two individuals) was also sampled as an outgroup for phylogenetic analysis (Table 1). All leaf samples were dried with silica gel until DNA extraction, and voucher specimens for representative individuals of each population were deposited in the SKK (the Ha Eun Herbarium, Sungkyunkwan University) and KNU (the Kyungpook National University Herbarium).

**Table 1:** List of populations and number of individuals of *R*. *crataegifolius* and *R*. *takesimensis* sampled in this study. For each population, haplotype and number of individuals in each haplotype are shown in parentheses.

Taxon/Population	Locality/Population code	Latitude (N)	Longitude (E)	Number	Haplotypes (number of individuals)
				of individual	
***Rubus crataegifolius***	**Korea (14 populations; 135 individuals)**				
Chungcheongnam-do	Taean-gun Gonam-myeon Nudong-ri/ND	36°45'035	126°40'470	3	H79(1), H80(1), H81(1)
Gangwon-do	Donghae-si Mukho-dong/DH	37°55'4706	129°11'843	13	H42(2), H44(1), H45(1), H54(5),
					H78(1), H88(1), H91(2)
	Inje-gun Muk-myeon Mt. Seolak/SA	38°05'711	128°27'025	10	H42(1), H55(3), H57(1), H67(1),
					H68(1), H69(1), H70(1), H71(1)
	Taebaek-si Mt. Hambaek/HB	37°16'12	128°92'007	9	H42(2), H57(1), H72(1), H75(1),
					H94(1), H96(1), H74(1), H89(1)
	Yeongwol-gun Yeongwol-eup Mt. Wantak/YW	37°20'527	128°56'146	1	H85(1)
	Yangyang-gun Seo-myeon Hangyeryeong/HG	38°09'764	128°40'614	1	H93(1)
Gyeonggi-do	Hwaseong-si Mt. Chilbo/CB	37°25'379	126°93'991	9	H42(4), H86(1), H87(1), H91(1), H95(1), H96(1)
	Incheon-si, Gangwha-gun Mt. Mani/GWH	37°60'738	126°44'383	1	H48(1)
	Incheon-si Baengyeong Island/BR	37°55'571	124°39'168	8	H40(1), H41(4), H97(2), H98(1)
Gyeongsangbuk-do	Daegu-si Namgu Mt. Bisul/BS	35°82'519	128°58'589	9	H42(1), H57(1), H82(1), H90(2),
					H99(1), H121(1), H122(2)
Gyeongsangnam-do	Sancheong-gun Samjang-myeon/SC	35°36'662	127°78'329	9	H47(1), H50(1), H51(1), H52(1),
					H53(1), H92(1), H104(1), H120(2)
	Yangsan-si Sangbuk-myeon Seokgye-ri/YS	35°41'665	129°10'005	6	H49(1), H53(1), H120(1), H105(3)
Jeollanam-do	Gwangyang-si Mt. Baekun/BU	35°03'981	127° 38'743	10	H42(1), H56(1), H76(1),
					H77(2), H105(5)
Jeju-do	Jeju-si Jochon-eup Geomun-oreum/JJ-GMO	33°27'163	126°42'463	10	H105(2), H111(3), H114(1),
					H125(2), H126(2)
	Jeju-si Jochon-eup Dang-oreum/ J-DANG	33°46'925	126°77'721	1	H116(1)
	Jeju-si Hallim-eup Geomeun-oreum/JJ-GOO	33°21'065	126°18'229	8	H105(4), H110(1), H121(2), H124(1)
	Jeju-si Ora2-dong Bangseon Bridge/JJ-BSG	33°25'110	126°31'193	7	H105(4), H121(1), H124(2)
	Seogwipo-si Pyosun-myeon Mt. Daerok/JJ-DRS	33°39'918	126°73'203	1	H122(1)
	Seogwipo-si Namwon-eup Sinrye-ri/JJ-SPpy	33°36'0948	126°59'703	12	H111(7), H113(1), H127(3), H128(1)
	Seogwipo-si Yongari-oreum/JJ-YAO	33°19'519	126°22'552	7	H105(1), H111(2), H112(1),
					H113(1), H126(1), H128(1)
***Rubus crataegifolius***	**Japan (6 populations; 51 individuals)**				
	Gochi Pref. Motoyamajo Shikoku/Shiko	33°73'18	133°60'28	1	H115(1)
	Hiroshima Pref. Hachiman Plateau/Hiro	37°58'41	132°75'31	8	H105(2), H107(6)
	Hokkaido Pref. Abashiri/BHD	43°76'24	144°26'78	1	H118(1)
	Ibaraki Pref. Ishioka, Ueso Pass/TK	36°48'030	140°08'356	8	H104(8)
	Miyagi Pref. Sendai Aoba-ku/Miya	38°15'11	140°49'34	8	H105(4), H106(4)
	Nagasaki Pref. Tsushima/DMD	34°09'15	129°15'06	1	H117(1)
	Nagasaki Pref, Unzen Spa/Naga	38°29'30	140°49'02	8	H107(2), H123(6)
	Oita Pref. Mt. Haneyama/Oita	33°23'36	131°13'41	8	H123(8)
	Yamagata Pref. Yamadera/Yama	32°75'52	130°29'32	8	H107(7), H123(1)
***Rubus crataegifolius***	**China (1 population; 8 individuals)**				
	Liaoning Shang Benxi/YR1	41°46'264	123°68'593	8	H60(3), H61(1), H62(1),
					H46(1), H84(2)
***Rubus crataegifolius***	**Russia (2 populations; 15 individuals)**				
	Promorsky Spasski (Spssk Dalny)/Rus 1	44°58'821	132°83'554	7	H42(1), H59(2), H66(1),
					H73(2), H129(1)
	Staraya Kamenka Lajov reserve/Rus 2	43°15'229	134°02'165	8	H63(1), H64(1), H 65(1), H83(5)
***Rubus takesimensis***	**Korea (12 populations; 106 individuals)**				
Ulleung Island	Ulleung-eup Dodong-ri/BRDF	37° 30'051	130° 53'495	8	H1(1), H10(1), H28(1),
					H29(2), H32(2), H33(1)
	Ulleung-eup Dodong-ri/DDHN	37° 48'597	130° 90'771	8	H15(1), H16(1), H38(2),
					H100(1), H103(1), H119(2)
	Ulleung-eup Sadong-ri/SDR	37° 29'683	130° 52'407	9	H9(2), H30(3), H37(1), H39(3)
	Ulleung-eup Dok-do Observatory/DDO	37° 28'784	130° 54'123	8	H1(3), H8(1), H13(1), H14(3)
	Buk-myeon Nari basin (Al-bong)/AB	37° 30'640	130° 51'860	11	H13(2), H21(1), H24(3), H25(1),
					H26(1), H29(2), H31(1)
	Buk-myeon Cheonbu-ri/SP	37° 31'850	130° 54'134	8	H1(2), H9(1), H24(1), H27(1),
					H32(1), H108(1), H109(1)
	Buk-myeon Chusan/CS	37° 31'920	130° 51'599	8	H1(1), H3(1), H4(1), H5(1), H29(1),
					H34(1), H43(1), H58(1)
	Ulleung-eup Jeodong Naesujeon/NSJ	37° 30'691	130° 54'030	8	H1(3), H14(2), H24(1), H32(2)
	Ulleung-eup Sadong-ri Jungnyeong/JR	37° 27'816	130° 52'251	13	H10(4), H11(1), H12(2),H18(1),H20(1),
					H22(1), H23(1), H24(1), H29(1)
	Seo-myeon Namseo-ri Taeharyeong/THR	37° 29'416	130° 49'620	7	H1(1), H7(1), H24(2), H25(1), H29(2)
	Seo-myeon Taeha-ri Seodal/SD	37° 30'212	130° 49'573	8	H1(2), H6(1), H17(1), H25(1),
					H29(2), H36(1)
	Ulleung-eup Dodong-ri/DDR16	37° 29'398	130° 54'727	10	H1(5), H2(1), H101(1), H102(1),
					H28(1), H35(1)
***Rubus trifidus***	**Korea (Outgroup)**				
**Gyeongsangnamdo**	Busan-si Namgu Yonghodong Yigidae	35°07'002	129°07'212	2	
**Total**				**317**	

### DNA isolation, PCR amplification, and sequencing

Total genomic DNA was extracted from dried leaves using a DNeasy plant mini kit (Qiagen, Valencia, California). After screening several noncoding regions of chloroplast DNA, we selected five highly variable intergenic spacers, i.e., *atp*H/*atp*I, *psa*I/*acc*D, *psb*D/*trn*T, *trn*G/*trn*S, and *trn*L/*trn*F [37,38]. PCR amplification was performed in a final volume of 50 μL, with each primer at a concentration of 1.0 pmol, along with 0.2 mM dNTPs, 2.5 mM MgCl_2_, and 1.25 U of Inclone^TM^ Taq DNA polymerase (InClone Biotech Co., Seoul, Korea). PCR conditions were an initial denaturation of 95°C for 2 min, 35 cycles of 95°C denaturation for 20 s, 56°C annealing for 40 s, 72°C extension for 1 min, and a final extension at 72°C for 5 min. PCR products were purified using the InClone^TM^ gel & PCR purification kit (InClone Biotech Co., Seoul, Korea). Sequencing reactions were performed for the purified PCR products using BigDye Terminator V3.1 Cycle Sequencing Kit (Applied Biosystems, Foster City, California) at the GenoTech Corp. (Daejeon, Korea).

### Phylogenetic analysis, haplotype network, genetic diversity and differentiation, and demographic change

All sequences were assembled and edited using Sequencher 4.2.2. (Gene Codes, Ann Arbor, MI, USA). Sequences were aligned using Clustal W version 2 [39], and minor adjustments were made manually using MacClade version 4.08 [40]. Based on the previous phylogenetic study of *Rubus* in Korea and phylogeographic analysis, *R*. *trifidus* was selected as an outgroup [33,34,36]. We performed maximum parsimony (MP) and maximum likelihood (ML) analysis using PAUP* 4.0b10 [41] and IQ-TREE version 1.5.5 [42], respectively, to determine the source population of *R*. *takesimensis* on Ulleung Island. All gaps were treated as missing data or coded as simple binary characters [43]. All characters were treated as unordered, and transformation of all characters was weighted equally. For ML analysis, the best-fit model “K3Pu+F+R2” was used to find the best scored ML tree using IQ-TREE program. For MP analysis, search options included heuristic searching with multistate taxa interpreted as uncertain, a simple stepwise addition of taxa, tree-bi-section reconnection (TBR) branch swapping, MulTrees options on, and zero branches collapsed. The bootstrap support (BS) value for each node was also calculated based on bootstrap analysis with 1,000 replicates using the same heuristic search options [44].

For DNA polymorphism, the number of haplotype, nucleotide diversity (π), gene diversity, and θπ were estimated by Arlequin version 3.5 [45]. To determine haplotype relationships between *R*. *crataegifolius* and *R*. *takesimensis*, we used the haplotype network program TCS version 1.21 [46]. Gaps were treated as missing data, and the connection limit excluding homoplastic changes was set to 95% in accordance with Hart and Sunday [47]. An analysis of molecular variance (AMOVA) implemented by Arlequin version 3.5 [45] was performed to estimate generic variation among and within populations. The AMOVA analyses were performed using three categories: (1) all samples from the two species; (2) the continental progenitor *R*. *crataegifolius*, using three groups from Korea, China and Russia, and Japan; and (3) the insular derivative, *R*. *takesimensis*. The significance of the variance components was calculated using 1,023 permutations. For demographic history, we used mismatch distribution analysis using Arlequin 3.5 [45] and DnaSP V. 5 [48]. The goodness-of-fit under a sudden expansion model was tested with the sum of squared deviations (SSD; [49]) and Harpending’s raggedness index (RI) [50]. A neutrality test, Tajima’s *D* [51], and Fu and Li’s test [52,53] were also performed to determine any evidence of recent range expansion. A parametric bootstrap approach with 1,000 replicates was used to test the observed mismatch distribution fit to the sudden expansion model. Lastly, we conducted PERMUT analysis [54] to test for the existence of geographical structures within populations of *R*. *takesimensis* on Ulleung Island and compared two coefficients for gene differentiation, *G*_*ST*_ and *N*_*ST*_, with 1,000 permutations. Nine populations with less than 3 individuals (i.e., ND, YW, HG, GWH, JJ-DANG, JJ-DRS, Shiko, BHD, and DMD) were excluded for the mismatch distribution, PERMUT analysis, and in the distribution of chloroplast haplotypes among populations.

## Results

### Phylogenetic relationship between *R*. *crataegifolius* and *R*. *takesimensis*

**Fig 1:**
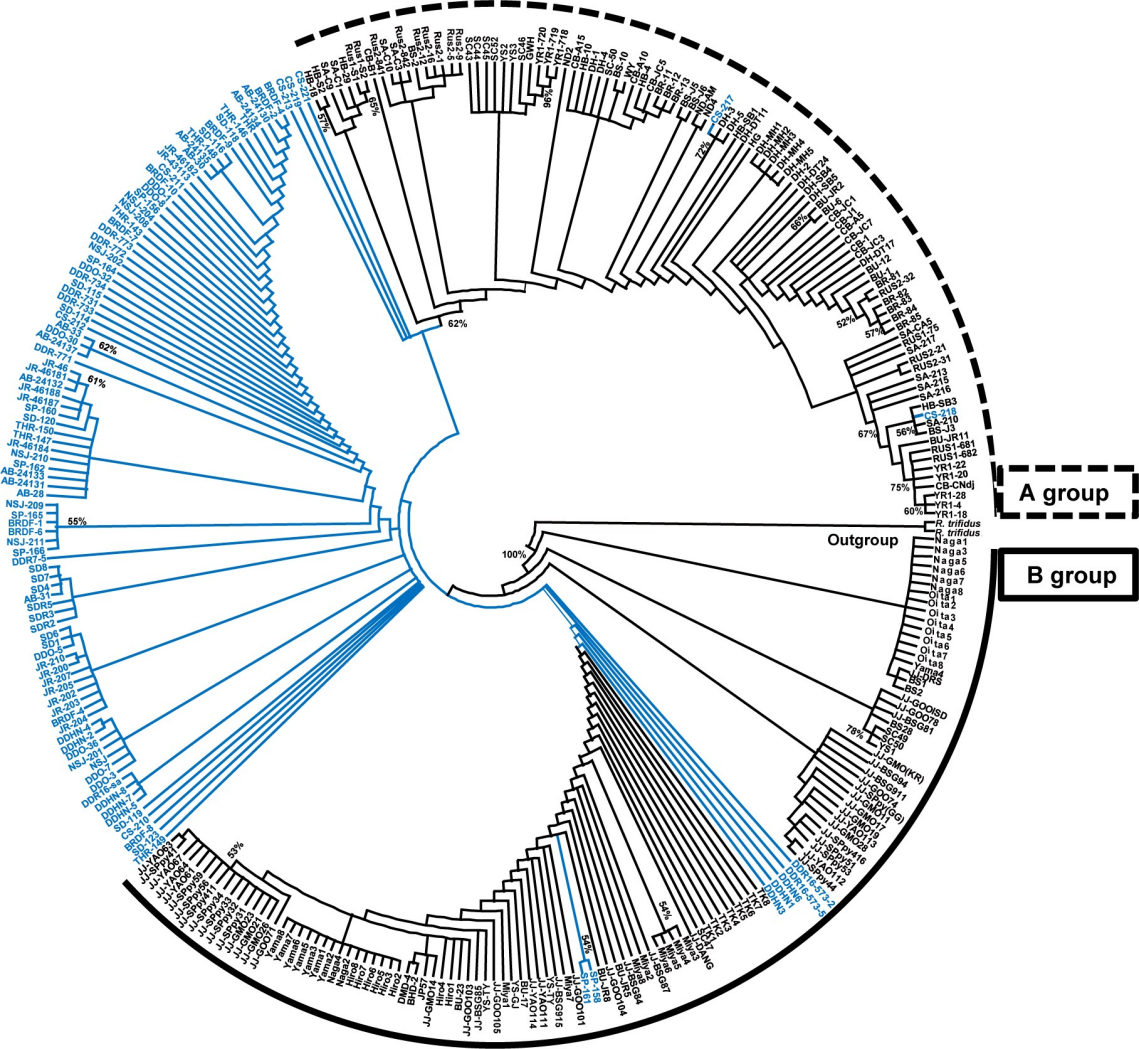
Phylogenetic tree. Strict consensus tree of 605 equally most parsimonious trees (TL = 296, CI = 0.67, and RI = 0.96), showing the relationship between the continental progenitor *R*. *crataegifolius* (in black) and the insular derivative *R*. *takesimensis* (in blue).

The combined aligned sites of the five intergenic spacers were 4,212 base pairs (bp). The *acc*D/*psa*I intergenic region was 706 bp long, including nine single nucleotide polymorphisms (SNPs, 1.2%) and four indels, while the *atp*H/*atp*I region was 576 bp (14 SNPs, 2.4%; one indel). The *psb*D/*trn*T region was 1,430 bp (30 SNPs, 2.0%; three indels), *trn*G/*trn*S was 560 bp (six SNPs, 1.07%; no indel), and *trn*L/*trn*F was 940 bp (five SNPs, 0.5%; three indels). Of 4,212 total aligned sites, 4,017 (95.37%) were constant, 57 (1.35%) were variable but parsimony-uninformative, and 138 (3.28%) were parsimony-informative characters. The MP analysis found 605 equally most parsimonious trees with a tree length (TL) of 296, a consistency index (CI) of 0.67, and a retention index (RI) of 0.96. Similar tree topology was found when the gaps were treated as either missing data or coded as binary characters, and thus, the strict consensus tree based on the treatment of gaps as missing data is shown (Fig 1). The ML tree (not shown) also showed essentially the same tree topology as the MP tree. Based on extensive sampling of *R*. *takesimensis* throughout the entire range on the island, our results were similar to what we showed earlier but provided new insights into the origin and evolution of *R*. *takesimensis* [36]. *Rubus takesimensis* emerged as non-monophyletic, with 12 individuals from Chusan (CS), Seokpo (SP), and Dodong (DDHN, DDR16) populations closely related to *R*. *crataegifolius* individuals. The group containing the most *R*. *takesimensis* individuals throughout the entire range of Ulleung Island shared the most recent common ancestor with populations of *R*. *crataegifolius* sampled from the northern part of the Korean peninsula, China, and the Russian Far East (hereafter called group A). The other group (hereafter called group B) included *R*. *crataegifolius*, primarily sampled from the southern part of the Korean peninsula, Jeju Island, and Japan. Within this group, southern populations sampled from Jeju Island (JJ-GMO, SPpy, and YAO), Korea, were closely related to the ones sampled from Hiroshima (Hiro) and Yamagata (Yama) prefectures in Japan. In group A, some individuals sampled from China (YR1), were closely related to the ones from Russia (Rus1), while other individuals were closely related to populations sampled from Korea (SC, YS, and SA). Some individuals from Russia (Rus2) were more closely related to the ones from the mountains of Seolak (SA) and Hambaek (HB) in the Gwangwoan-do province of Korea. It was noticeable that some individuals of *R*. *takesimensis* were more closely related to *R*. *crataegifolius* than to other conspecific populations on the island; three of eight individuals sampled from Dodong (DDHN), two of ten individuals sampled from Jeodong (DDR16), and two of eight individuals from Seokpo (SP). Furthermore, five of eight individuals of *R*. *takesimensis* from the Chusan population (CS) were closely related to group A of *R*. *crataegifolius*; two individuals, 217 and 218, were closely related to populations sampled from Gwangwon-do province (DH) and Gwangwon-do/Gyeongsangbuk-do province (HB, SA, and BS), respectively.

### Genealogical relationships among haplotypes

**Fig 2:**
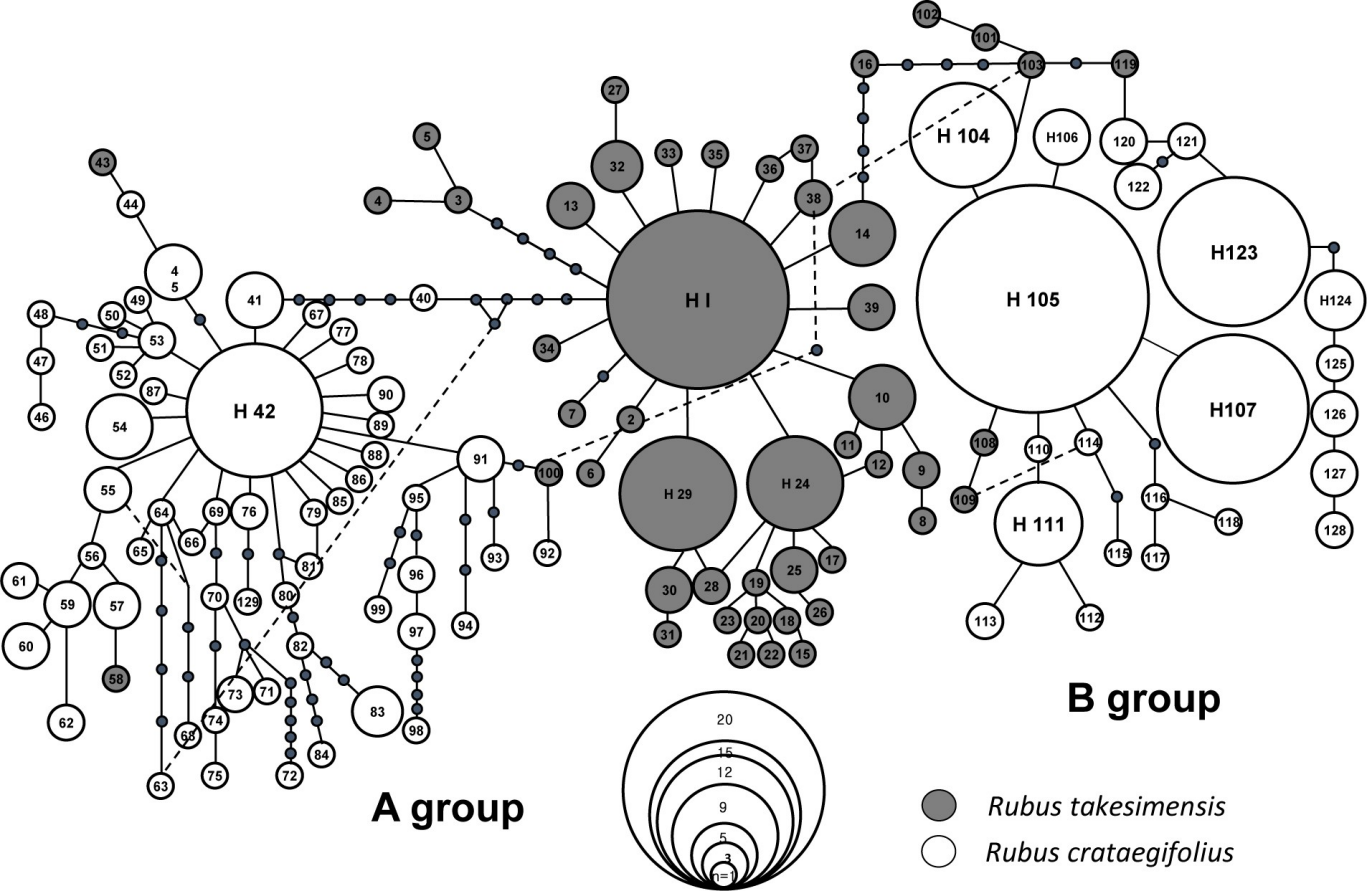
TCS haplotype network. TCS haplotype network of 129 haplotypes found in *R*. *crataegifolius* (81 haplotypes in open circles) and *R*. *takesimensis* (48 haplotypes in closed circles). Dots indicate hypothetical missing intermediate haplotypes. The size of each circle is proportional to the relative haplotype frequency. Two major haplotype groups (A and B) of *R*. *crataegifolius* are shown.

The TCS haplotype network (Fig 2) showed essentially the same relationships as shown in the phylogenetic tree (Fig 1). We found a total of 129 haplotypes between the continental progenitor *R*. *crataegifolius* and the insular-derived *R*. *takesimensis*. Of 129 haplotypes, 81 and 48 haplotypes were found exclusively in *R*. *crataegifolius* and *R*. *takesimensis*, respectively. Most haplotypes were found at low frequency, and no haplotype was shared between the two species pair. For the insular derivative *R*. *takesimensis*, most haplotypes, except for individuals sampled from Chusan (H3, H4, and H5), were connected by one mutational step from major haplotypes, suggesting recent origins from the ancestral haplotype. Haplotypes H1 (18 individuals), H24 (eight individuals), and H29 (ten individuals) showed high frequency in *R*. *takesimensis* on Ulleung Island.

In most cases, each haplotype was represented by one individual, but rarely by two to three individuals. Eight haplotypes, each one represented by a single individual, were found in the Chusan population (CS). Specifically, haplotypes H43 and H58 were more closely related to the haplotype found in *R*. *crataegifolius* sampled from the northern part of the Korean peninsula. Two haplotypes found in Dodong-ri (DDHN), H103 and H119, were also closely related to *R*. *crataegifolius* sampled from the southern part of the Korean peninsula. Of seven haplotypes found in Seokpo (SP), two haplotypes (H108 and H109) were closely connected to haplotypes of *R*. *crataegifolius* sampled from Jeju Island, Korea (H105 and H114). These cases represent several rare haplotypes on the island more closely related to those of *R*. *crataegifolius*, indicating their independent origins from different continental source areas.

In the case of the continental progenitor *R*. *crataegifolius* shown in the phylogenetic tree, we found two divergent clusters: group A included major haplotypes sampled from the northern part of the Korean peninsula, while group B included major haplotypes from the southern part of the Korea peninsula, Jeju Island, and Japan. Higher haplotype diversity was found in populations sampled from the Korean peninsula compared with the ones in Japan. The population sampled from Baekryeong Island (BR) in the Yellow Sea contained two divergent haplotype groups, one with H40 and H41, and the other with H97 and H98, and two somewhat divergent haplotypes (H40 and H41) were connected to the most common haplotype (H1) of *R*. *takesimensis*. The most common haplotype within this group was H42 (12 individuals), which was found in Gwangwon-do province (HB and DH), Gyeonggi-do province (CB), and Gyeongsangbuk-do province (BS) in Korea. Unlike haplotypes found in *R*. *takesimensis*, haplotypes found in this group A were connected by one to seven missing or inferred haplotypes, suggesting the divergent nature of the haplotype group. Of the five haplotypes found in one population from China (YR1), three (H60, H61, and H62) were connected to the haplotype H59 sampled from Russia (Rus1), while the remaining two (H46 and H84) were related to the southern part of the Korean peninsula (H47 from SC and H82 from BS). Most haplotypes found in Russia, except for two haplotypes, were connected to the ones from the northern part of the Korean peninsula (SA and BS). Two haplotypes (H43 and H58) of *R*. *takesimensis* sampled from Chusan (CS) on Ulleung Island were connected to the ones from Gangwon-do province (H44 from DH and H57 from HB, respectively).

The group B included haplotypes found primarily in the southern part of the Korean peninsula (Gyeongsangnam-do and Jeollanam-do province), Jeju Island, and Japan. The most common haplotype was H105 (23 individuals), which occurred in the southern part of Korea (YS and BU), Jeju Island, and Japan (Hiro and Miya). The haplotypes found in *R*. *takesimensis* were connected to these southern populations via several missing haplotypes. The haplotype found in Jeju Island (H105) showed regional sharing across the Halla Mountains between the northern and southern parts of the island. Two haplotypes (H108 and H109) of *R*. *takesimensis* found from Seokpo (SP) on Ulleung Island were connected to H105 and H114 found on the northern part of Jeju Island.

### Diversity and distribution of chloroplast haplotypes among populations

**Fig 3:**
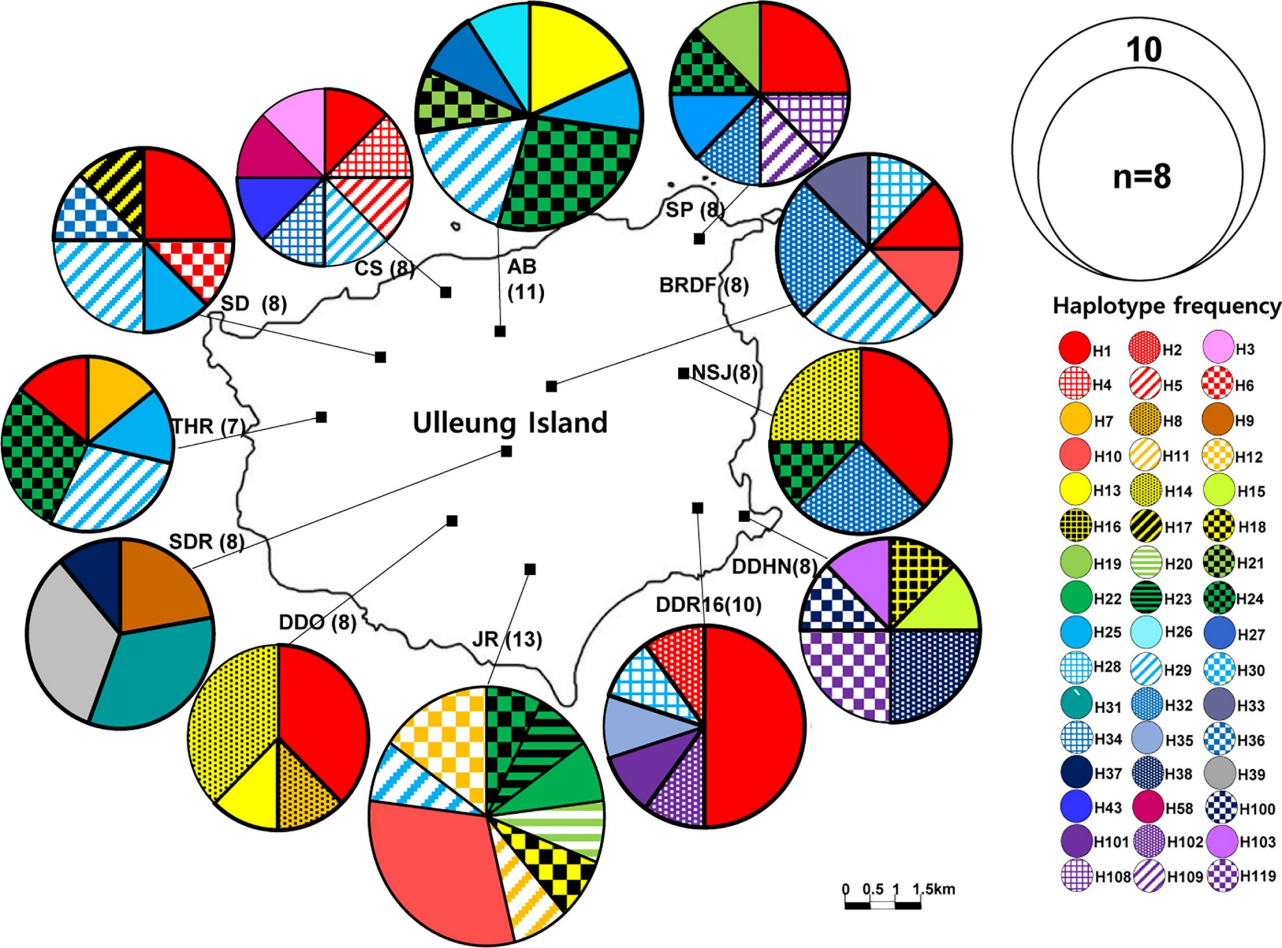
Map of haplotypes found in *R*. *takesimensis*. Distribution of each haplotype and its frequency found in *R*. *takesimensis* from Ulleung Island. The size of each circle is proportional to the population size. Different colored and patterned portions in each pie chart represent haplotype frequencies.

**Fig 4:**
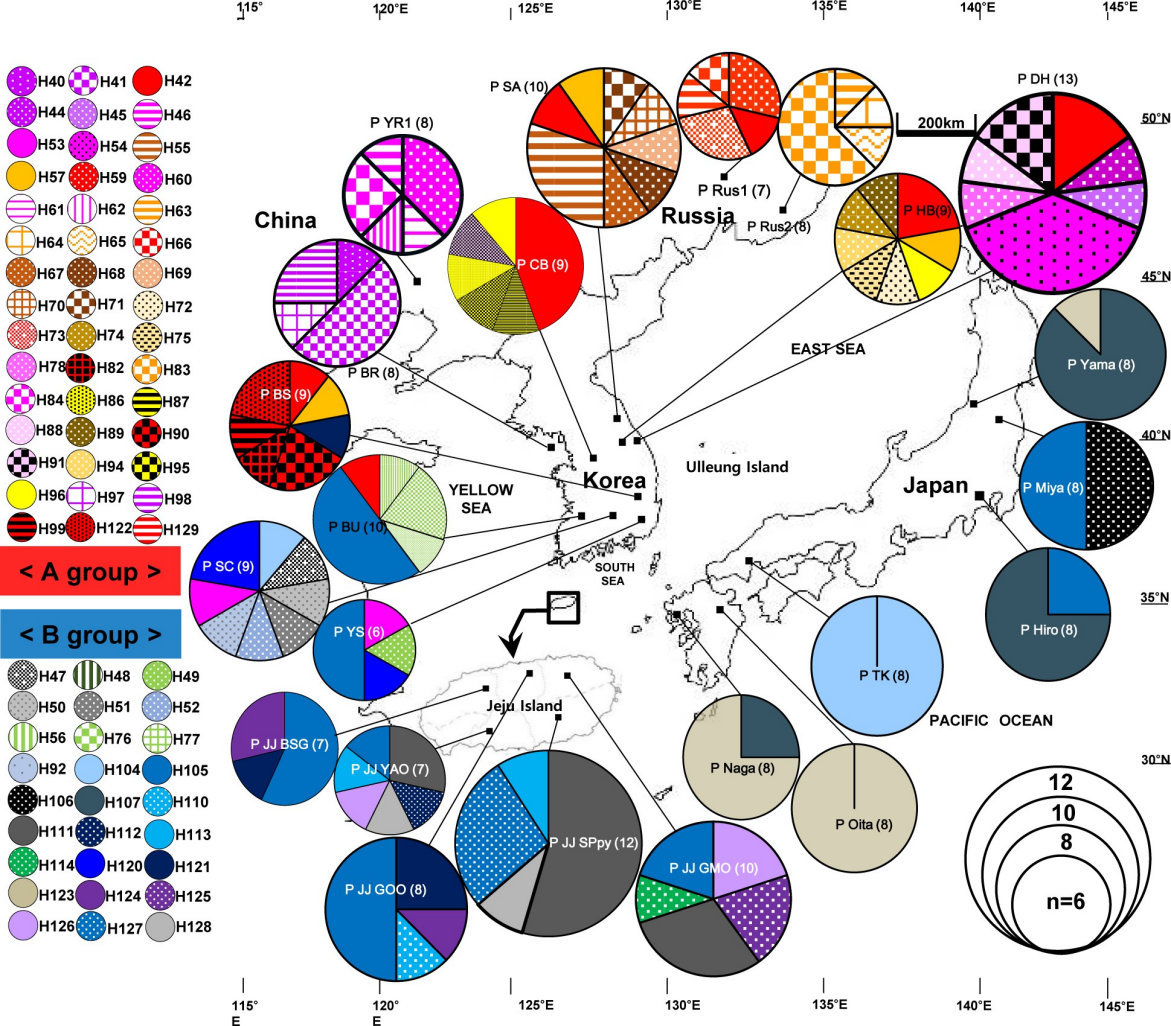
Map of haplotypes found in *R*. *crataegifolius*. Distribution of 81 haplotypes found in *R*. *crataegifolius*. The size of each circle is proportional to the population size. Different colored and patterned portions in each pie chart represent haplotype frequencies.

The number of haplotypes found in each population of *R*. *takesimensis* on Ulleung Island ranged from four (DDO, NSJ, and SDR) to nine (JR) (Table 1 and Fig 3). Some populations sampled from the northern part of the island, such as the Nari Basin (AB; seven haplotypes), Chusan (CS; eight haplotypes), and Seokpo (SP; seven haplotypes) harbored diverse haplotypes. In addition, some haplotypes (e.g., H1, H24, and H29) occurred widely in different populations, whereas others (e.g., H2, H3, H4, H5, among others) were narrowly restricted to specific populations. A total of 81 haplotypes were found in *R*. *crataegifolius*, and two divergent groups were observed (Fig 4). Group A included populations primarily sampled from the mainland part of the Korean peninsula, Russia, and China. For the 14 populations sampled from Korea, the number of haplotypes per population ranged from three (JJ-BSG) to eight (HB, SA, and SC). Of the 14 populations, six populations (BR, CB, BS, SA, HB, and DH) contained primarily group A haplotypes, while the remaining three from the southern peninsula (BU, SC, and YS) and four populations from Jeju Island contained the group B haplotypes. For the three populations sampled from Russia and China, the number of haplotypes ranged from four (Rus2) to five (Rus1 and YR1), and they are closely related to the ones from the northern part of the Korean peninsula.

Contrary to group A, with its diverse haplotypes, group B included populations primarily from the southern part of the Korean peninsula and Japan. For the six populations sampled from Japan, all but two populations (Oita and TK) contained two haplotypes, showing highly reduced haplotype diversity. Two populations, Oita and TK, contained H123 (shared with the nearby Naga population) and H104 (specific to the TK population), respectively.

### Genetic diversity and structure between *R*. *crataegifolius* and *R*. *takesimensis*

With regard to the partitioning of genetic variations between the two taxa (the continental progenitor *R*. *crataegifolius* and the insular derivative *R*. *takesimensis*), the majority of variations (73%) existed within populations, while approximately 27% of the variations existed among populations (Table 2). Within a continental progenitor species *R*. *crataegifolius*, slightly more than half of the total variations (i.e., 57%) occurred among populations, while the remaining 17% and 26.5% of the variations existed among countries and within populations, respectively. In the case of the insular derivative species *R*. *takesimensis*, total variations were partitioned more-or-less equally among populations (57%) and within populations (43%).

**Table 2:** Summary of analyses of molecular variance (AMOVA) in *R*. *crataegifolius* and *R*. *takesimensis*, showing degree of freedom (*df*), sum of squares (SS), variance components, and the total variance contributed by each component (%) and its associated significance (*n* = 1,023 permutations).

Taxon	Source of Variation	*df*	SS	Variance Components	Total Variance (%)	Fixation Indices	*P* value
(a) *R*. *crataegifolius* and *R*. *takesimensis*	Between taxa	1	806.776	0.02925	0.10	F_CT_ = 0.00097	0.75171±0.01677
	Among populations	2	868.650	8.10493	26.91	F_SC_ = 0.26939	0.00±0.00
	Within populations	292	6418.584	21.98145	72.99	F_ST_ = 0.27010	0.00±0.00
(b) *R*. *crataegifolius*	Among Country (Korea/Japan/Others)	2	856.166	6.06414	16.92	F_CT_ = 0.16922	0.00880±0.00320
	Among populations	39	3973.384	20.27419	56.57	F_SC_ = 0.68097	0.00±0.00
	Within populations	152	1443.769	9.49848	26.50	F_ST_ = 0.73495	0.00±0.00
**(**c) *R*. *takesimensis*	Among populations	14	861.537	7.94952	56.86		
	Within populations	90	542.911	6.03234	43.14	F_ST_ = 0.56856	0.00±0.00

In the case of the genetic diversity between continental progenitors and insular derivative species, we found significantly higher diversity statistics in *R*. *crataegifolius* than in *R*. *takesimensis*: the number of haplotypes (81 vs. 48), nucleotide diversity (π) (0.015951 vs. 0.007045), and θπ (65.0013 vs. 27.67857) (Table 3). However, we cautiously interpreted these diversity statistics given the unequal sample size between two species: 209 individuals in *R*. *crataegifolius* vs. 106 individuals in *R*. *takesimensis*.

**Table 3:** Summary of genetic diversity statistics of the continental progenitor *R*. *crataegifolius* and the insular derivative *R*. *takesimensis* based on chloroplast DNA noncoding sequences.

Species	No. of Individuals	No. of Haplotype	Nucleotide Diversity (π)	Gene Diversity	θπ
*R*. *crataegifolius*	209	81	0.015951 ± 0.007642	0.9677 ± 0.0049	65.0013 ± 31.14162
*R*. *takesimensis*	106	48	0.007045 ± 0.003443	0.9500 ± 0.0017	27.67857 ± 13.52662
Total	315	129			

### Demographic change and geographical structure

**Fig 5:**
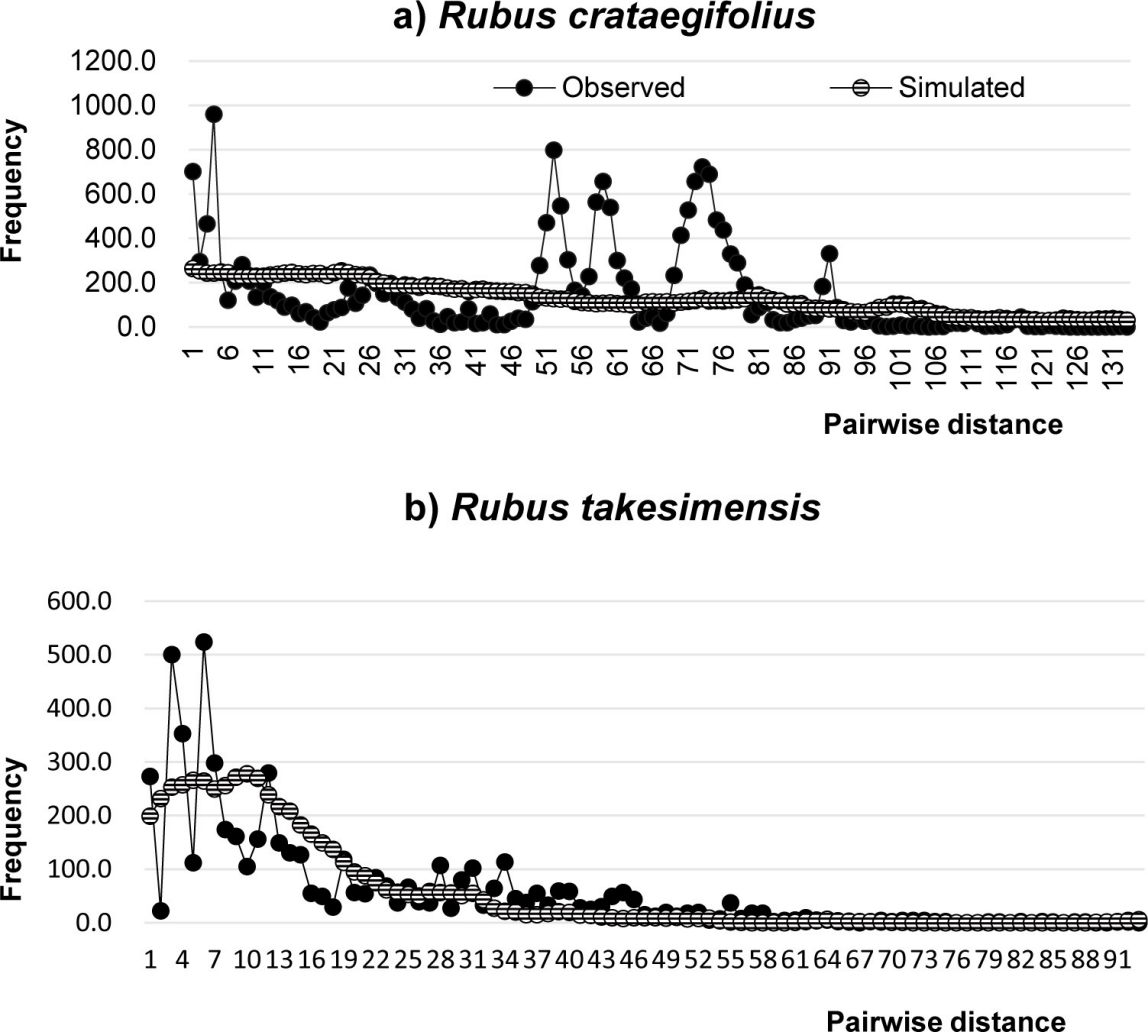
Mismatch analysis. Mismatch distribution analysis inferring the demographic history of *R*. *crataegifolius* (a) and *R*. *takesimensis* (b). The x-axis represents the number of pairwise differences, while the y-axis represents the relative frequencies of pairwise comparisons.

Mismatch distributions showed that *R*. *crataegifolius* had multimodal distributions with an SSD of 0.007 (P<0.05) and RI of 0.0074 (P<0.05) (Fig 5 and Table 4). Also, populations sampled from *R*. *crataegifolius* on the Korean peninsula only showed multimodal distributions. Mismatch distributions of *R*. *takesimensis* were also multimodal, with an SSD of 0.0149 (not significant) and an RI of 0.0355 (P<0.05). Therefore, it is likely that the population of *R*. *takesimensis* did not undergo recent population expansion or a bottleneck after introduction to Ulleung Island [53,54]. Demographic analysis showed negative values for Fu’s *Fs* and Tajima’s *D* test (Table 4), with significant values for *R*. *crataegifolius* only (*Fs* = -2.75529, P <0.05). The Harpending’s raggedness index (RI) derived from the mismatch distribution was significant (Table 4). In *R*. *takesimensis* on Ulleung Island, the total genetic diversity (*h*_*T*_ = 0.885), based on cp DNA data across all populations, was much higher than the average within the population diversity (*h*_*S*_ = 0.701). The *G*_*ST*_ value (0.208) of *R*. *takesimensis* on Ulleung Island was higher than its corresponding *N*_*ST*_ (0.139), suggesting no significant genetic structure in *R*. *takesimensis* on Ulleung Island.

**Table 4:** Neutrality and population expansion tests for *Rubus crataegifolius* and *R*. *takesimensis*.

Taxa	Tajima’s *D*	Fu’s *Fs*	Sum of squared deviation (SSD)	Harpending’s raggedness index (RI)
*R*. *crataegifolius*	-1.46400 ^ns^	-2.75529 *	0.0070*	0.0074*
*R*. *takesimensis*	-1.61222 ^ns^	-2.09349 ^ns^	0.0149 ^ns^	0.0355*

Tajima’s *D*, Fu’s *Fs* statistic, Sum of squared deviation (SSD), and Harpending’s Raggedness index (RI) with significance derived from 10,000 simulations.

Asterisks denote significant differences:

* P < 0.05

Ns: Not significant: P > 0.10, 0.10 > P > 0.05

## Discussion

### Multiple origins of *R*. *takesimensis* and patterns of anagenetic speciation on Ulleung Island

This study represents the first attempt to utilize chloroplast DNA based on extensive sampling (315 individuals) and sequence data (aligned sequence of 4,212 bp) to characterize the genetic consequences of anagenetic speciation in one endemic species-rich group, Rosaceae, on Ulleung Island, Korea. Previous studies were limited to the Sapindaceae (i.e., *Acer takesimensis* and *Acer okamotoanum*) and the Apiaceae (*Dystaenia takesimana*), primarily utilizing nuclear markers (i.e., SSRs, AFLPs, and nrDNA ITS) with complimentary use of a chloroplast marker (*trn*L-F) [23–25,55]. It may not be possible to make direct comparisons between our study, which was based on maternally inherited cpDNA and thus focused on seed-mediated gene flow, and previous studies, which primarily utilized biparentally inherited nuclear DNA. However, the general trend and pattern found in this study could be compared and evaluated as a proxy of nuclear genetic diversity, given the unusually high level of chloroplast diversity in *Rubus* species.

First of all, we found that endemic species of *R*. *takesimensis* on Ulleung Island are not the result of a single colonization event. Rather, several geographically diverse continental source populations were likely involved in the origin of *R*. *takesimensis*. As shown in the phylogenetic tree (Fig 1) and the haplotype network (Fig 2), a major group of *R*. *takesimensis* accessions were closely related to the group containing *R*. *crataegifolius* accessions primarily sampled from the northern part of the Korean peninsula, while rare accessions from Dodong (DDR16 and DDHN) and Seokpo (SP) most likely originated from the southern part of the Korean peninsula, Jeju Island, and Japan. Furthermore, even within a population, especially Chusan (CS) in the north-facing part of the island, we found evidence for a few accessions originating separately from the Korean peninsula. These populations are located either nearby a harbor used for commercial fishing and passenger ferries or near typical bird nesting sites, thus increasing the likelihood of anthropogenic or natural independent colonization events from different source populations. It seems less likely though that human-mediated colonization is responsible for the origin of some accessions in those populations because the archaeological evidence indicates that the island has been inhabited since the 1^st^ millennium BC, with the first confirmed historical reference for the year of 512 CE [56–58]. Considering the geographical proximity of Ulleung Island to possible source areas, i.e., 137 km from the Korean peninsula and approximately 240 km from Oki Island, Japan, multiple introductions may not be exceptional [27]. Pfosser et al. [55], based on one noncoding cpDNA and AFLPs, initially raised the possibility of multiple origins for *A*. *okamotoanum* on Ulleung Island, but later suggested a single origin from the continental progenitor *A*. *mono* based on microsatellite or SSR markers [24]. This discrepancy based on different types of molecular markers may be caused by incomplete lineage sorting and shared ancestral polymorphism and/or gene introgression between closely related species, as well as limited genetic information revealed by low evolutionary rate of the plastid genome [59–62]. It may be necessary to use highly variable SSRs or genome-wide SNPs to confirm our current results. Our earlier study [36] also raised the possibility of colonization of the most common haplotype in *R*. *takesimensis* back to the southern part of the Korean peninsula. However, current studies (JY Yang, unpublished data) involving extensive sampling from southern areas did not find any haplotype sharing between continental and insular species, suggesting Ulleung Island as a primary sink area for plant colonization despite its geographical proximity to the continent. Nevertheless, this study raises the possibility of multiple introductions for Ulleung Island endemics from nearby source areas, and additional investigations on diverse phylogenetic lineages with different seed dispersal modes will be helpful to determine the evolutionary history of each group.

Based on the few available studies of anagenetically derived species on Ulleung Island, some emerging patterns of the genetic consequences include a clear genetic distinction between continental progenitor and island-derived species and high levels of genetic variation within the island population with no geographic genetic partitioning [23,24,63]. Furthermore, a recent comprehensive review confirmed the same patterns, i.e., clear genetic differences between continental progenitors and insular derivative species, different levels of genetic variation depending on species, and no geographical partitioning of the genetic variation [26]. Based on the chloroplast DNA noncoding sequences, we found a significant reduction in haplotype number and nucleotide diversity in *R*. *takesimensis*, the insular derivative, compared with its continental progenitor *R*. *crataegifolius* (Table 3). This finding, however, could be due to uneven sampling between the two species. *Rubus takesimensis* harbors unique and diverse chlorotypes despite its occurrence in a young (<1.8 million years old) and small isolated island (a total area of 73 km^2^). Although we used five highly variable noncoding regions of cpDNA in our study, this level of haplotype diversity (i.e., 48 chlorotypes) contrasts with that of other endemic species, such as *Fagus multinervis* (one haplotype based on the *psb*A-*trn*H noncoding region) [64] and *Sedum takesimense* (two haplotypes based on the *trn*L-F noncoding region) [65]. Therefore, we suggest that *R*. *takesimensis* is genetically unique and contains comparable genetic diversity relative to its continental progenitor species.

### Factors affecting patterns of genetic variation in *R*. *takesimensis*

As suggested by Takayama et al. [26], several factors, such as population size, breeding system, and species age can contribute to the genetic consequences of anagenetically derived species on Ulleung Island. The age of *R*. *takesimensis* on Ulleung Island was estimated to be 1.7 ± 3.02 million years old, suggesting its origin soon after the formation of the island (JY Yang, unpublished data). In addition, species of *Rubus* are known for bird-mediated seed dispersal, employ diverse modes of reproduction (sexual and asexual), and play as early forest succession frontiers. When a single individual of *Rubus* is transplanted into a bare and open area, it expands quickly as a population with asexual creeping stems and roots in less than five years and undergoes sexual reproduction with the production of flowers and fruits (JY Yang, personal observation). In addition, *Rubus* species can utilize both asexual (obligate apomixes to facultative apomixes) and sexual (obligate sexuality) reproductive modes as the population expands [66]. As a consequence, these strategies allow fast formation and stabilization of a population via asexual reproduction, and maintain high genetic diversity from sexual reproduction [67–72]. High levels of genetic variation in vegetatively propagated species have been reported in the North American quaking aspen, *Populus tremuloidess* [73]. Sarhanova et al. [74] pointed out that anthropogenic disturbances, such as road construction, deforestation, and landscape changes, lead to the fast evolution of bramble species in Europe. *Rubus takesimensis* can vigorously expand its habitat to open areas as frontier plants, accumulate mutations in its populations, and receive a continuous supply of new genetic materials from continental progenitors through migrating birds. A combination of these factors, i.e., large population size, dual mode of reproduction, bird-mediated seed dispersal, and the long presence on the island likely resulted in high genetic diversity and no geographical genetic structuring in *R*. *takesimensis*.

As for bird-mediated seed dispersal, variations in fruit and seed size have potential effects on seed dispersal. For example, it has been demonstrated that frugivory of many passerine birds is limited by their gape width [75,76]. Birds also prefer fruits with low seed to fruit ratios [75,77]. Moreover, large seeds are often regurgitated after a relatively short time, whereas small seeds pass through the gut and are defecated after longer periods [78]. Therefore, small fruits and small seeds are more likely to be dispersed over long distances [79]. Brambles are typical bird-dispersed plants with a wide spectrum of dispersers [80,81]. European blackberries are mainly dispersed by frugivorous passerines [76,80]. Also, Jordano [80] and Rejmanek [81] reported that migrant birds are the main seed dispersers of Spanish populations of *R*. *ulmifolius* and a southern Spanish population of blackberry. The Ulleung and Dok-do Islands are known as refuge islands for migratory birds [82]. In the case of avian fauna on Ulleung Island, the Japanese wood pigeon (*Columba janthina*) is one notable bird species, and several other birds have also been reported; the black-tailed gull (*Larus crassiroostris*), the russet sparrow (*Passer rutilans*), the barn swallow (*Hirundo rustica*), and the oriental greenfinch (*Carduelis sinica*) [82]. Other permanent residents on Ulleung Island include the brown-eared bulbul (*Hypsipetes amaurotis*), the great tit (*Parus major*), the pheasant (*Phasianus colchicus*), and the blue rock thrush (*Monticola solitarius*) [82]. Although a total of 112 bird species in 38 families and 16 orders have been recorded [83], little is known regarding which species feed on *R*. *takesimensis* on Ulleung Island. Considering the habit of *R*. *takesimensis* (i.e., subshrubs), it is plausible that species belonging to the order Passeriformes could feed on aggregate druplets of *R*. *takesimensis*. The Passeriformes, suborders Passeri are small or medium-sized birds, mostly insectivorous or omnivorous, and known raspberry feeding birds include the catbird, the mocking bird, the robin, the tanager, the thrasher, the turkey, and the warbler [76]. The Passeriformes, *Carduelis sinica* (oriental green finch), *Passer rutilans* (russet sparrow), and *Zosterops japonicas* (warbling white-eye) are found on Ulleung Island. *Carduelis sinica* is assumed to be a permanent resident on the island, while *Zosterops japonicas* occur in southern coastal areas of the Korean peninsula, Jeju Island, and Ulleung Island. *Passer rutilans*, a winter migratory bird that visits southern parts of the Korean peninsula for the summer, occurs much more broadly in eastern Asia, Sakhalin, Jeju Island, and Ulleung Island. Considering some *R*. *takesimensis* haplotypes (e.g., Chusan) are closely related to *R*. *crataegifolius* haplotypes from the northern part of the Korean peninsula, China, and Russian Far East and others (i.e., Dodong, Jeodong, and Seokpo) to the southern part of the Korean peninsula, Jeju Island, and Japan, it is conceivable that such migratory birds are responsible for introducing a diverse pool of *R*. *crataegifolius* haplotypes to Ulleung Island, eventually evolving into *R*. *takesimensis*.

### Haplotype diversity in continental *R*. *crataegifolius*

For the patterns of genetic variation in the continental progenitor species *R*. *crataegifolius*, we found that populations sampled from the inland part of the Korean peninsula showed much higher haplotype diversity compared with those from Japan. In addition, haplotype composition among the populations from Japan was very simple, i.e., just one fixed haplotype, or up to two closely related haplotypes (except for H123). In Korea, *R*. *crataegifolius* frequently occurs in lowland open habitats and along mountainous trail edges all over the Korean peninsula, while it is rarely found in the mountainous areas of Japan. Considering the wide geographic natural distribution of *R*. *crataegifolius* in Japan, it was interesting to find very low genetic variation within populations and differentiation among populations compared with conspecific populations on the continent. Several factors (e.g., breeding system, demographic history, gene dispersal, among others) likely contribute to neutral genetic diversity within populations and genetic differentiation among populations. In particular, vegetative propagation and the selfing rate have profound outcomes for the genetic diversity of species [84,85]. In the genus *Rubus*, it has been demonstrated that sexual *R*. *ideanus* has much higher genetic variation than apomictic *R*. *nessensis* [86]. Although the mode of reproduction in populations of *R*. *crataegifolius* is unknown, it is conceivable that populations in Korea are sexual reproducing diploid or facultative apomictic, while those in Japan are predominantly obligate apomictic. The breeding system of *R*. *crataegifoloius* and other possible factors for shaping genetic variation and differentiation requires further investigation.

Given the rarity of sharing certain haplotypes between and among populations, it was noticeable to find the haplotype H105, which occurs commonly in the southern part of the Korean peninsula (i.e., Gyeongsangnam-do, Jeollanam-do, and Jeju-do Provinces), is shared with populations (northern Miya and southern Hiro) throughout the Japanese archipelago. Three derived haplotypes from H105 by one mutational step, i.e., H104, H106, and H107, were shared among populations (H107) or unique (H104 and H106) to a specific population. Therefore, the sharing of haplotype H105 between southern Korea and Japan may suggest a recent migration of *R*. *crataegifolius* from the Korean peninsula to the Japanese archipelago via land bridges, which existed during the last glacial maximum (LGM; the period between 25,000 and 18,000 before present) and served as a dispersal corridor during that time [36,87–89]. Alternatively, it is also conceivable that the haplotype H105 of *R*. *crataegifolius* colonized Japan via long-distance dispersal most likely by birds during or even before the Quaternary climatic oscillation, and survived during the LGM. Given all other haplotypes are confined within each region (the Korean peninsula and the Japanese archipelago), the Korean/Tsushima Strait served as an effective barrier to seed-mediated gene flow [90–93].

## Conclusions

We provided the first comprehensive results regarding the genetic consequences of the anagenetic speciation of *R*. *takesimensis* on Ulleung Island from the continental progenitor *R*. *crataegifolius* based on chloroplast DNA sequences. The insular derivate endemic species, *R*. *takesimensis* on Ulleung Island, originated multiple times from diverse geographical source populations. A distinct pool of chlorotypes was found between the progenitor and derivative species, suggesting significant genetic divergence between the two species. A highly diverse haplotype composition within the population and a lack of significant geographic structuring of *R*. *takesimensis* were found. Two major groups of chlorotypes in *R*. *crataegifolius* were found, with high diversity in the mainland continent populations than in the Japanese archipelago. Based on the results of anagenetic speciation of *R*. *takesimensis*, it seems necessary to test rigorously the monophyly of diverse insular endemic lineages on Ulleung Island based on extensive sampling strategy (i.e., covering diverse geographical source areas for continental progenitor species and anagenetically derived insular populations) and sequence data (i.e., including genome-wide SNPs and fastly evolving genes) and compare and contrast the emerging patterns in the genetic consequences of anagenetic speciation shaped by different types of molecular markers. In addition, the discovery of cryptic species on oceanic archipelagos in collaboration with others in the botanical disciplines is critical to the discovery and conservation of the subtle diversity in oceanic island flora [94].

## Supporting information

S1 Table Accession numbers. Accession numbers for haplotype sequences of *Rubus crataegifolius* and *R*. *takesimensis*.
(DOCX)

S2 Table Variable sites in Rubus takesimensis. Variable sites found in *Rubus takesimensis* identifying 48 haplotypes.
(XLSX)

S3 Table Variable sites in Rubus crataegifolius. Variable sites found in *Rubus crataegifolius* identifying 81 haplotypes.
(XLSX)
