# Supplementary material for: Multiple origins and the population genetic structure of Rubus takesimensis (Rosaceae) on Ulleung Island: Implications for the genetic consequences of anagenetic speciation
Source: PLoS One. 2019 Sep 19;14(9):e0222707. doi: 10.1371/journal.pone.0222707 (PMC6752786; doi:10.1371/journal.pone.0222707)
Supplement: S1 Table — Accession numbers for haplotype sequences of Rubus crataegifolius and R. takesimensis. (DOCX) [file pone.0222707.s001.docx]

***Supplementary Material***

**Multiple origins and the population genetic structure of *Rubus takesimensis* (Rosaceae) on Ulleung Island: implications for the genetic consequences of anagenetic speciation**

**Ji Young Yang,^1^ Jae-Hong Pak,^1 *^, Masayuki Maki,^2^ and Seung-Chul Kim,^3*^**

**Correspondence: Jae-Hong Pak:** [**jhpak@knu.ac.kr**](mailto:jhpak@knu.ac.kr)**;**

**Seung-Chul Kim:** [**sonchus96@skku.edu**](mailto:sonchus96@skku.edu)

**Supplementary Tables**

**Supplementary Table 1**. Accession numbers for haplotype sequences of *Rubus crataegifolius* and *R*. *takesimensis*

| Chloroplast intergenic region | *Rubus takesimensis* | *Rubus crataegifolius* |
| --- | --- | --- |
| *acc*D/*psa*I | MK948614-MK948621 | MK948679-MK948693 |
| *atp*H/*atp*I | MK948622-MK948635 | MK948694-MK948708 |
| *psb*D/*trn*T | MK948636-MK948656 | MK948709-MK948754 |
| *trn*G/*trn*S | MK948657-MK948670 | MK948755-MK948772 |
| *trn*L/*trn*F | MK948671-MK948678 | MK948773-MK948784 |

***Supplementary Material***

**Multiple origins and the population genetic structure of *Rubus takesimensis* (Rosaceae) on Ulleung Island: implications for the genetic consequences of anagenetic speciation**

**Ji Young Yang,^1^ Jae-Hong Pak,^1 *^, Masayuki Maki,^2^ and Seung-Chul Kim,^3*^**

**Correspondence: Jae-Hong Pak:** [**jhpak@knu.ac.kr**](mailto:jhpak@knu.ac.kr)**;**

**Seung-Chul Kim:** [**sonchus96@skku.edu**](mailto:sonchus96@skku.edu)

**Supplementary Tables**

**Supplementary Table 2**.

Variable sites found in *Rubus takesimensis* identifying 48 haplotypes.

**Supplementary Table 3**.

Variable sites found in *Rubus crataegifolius* identifying 81 haplotypes.
